# Investigation of Caring Behavior and Caring Burden and Their Associated Factors among Nurses Who Cared for Patients with COVID-19 in East Guilan, the North of Iran

**DOI:** 10.1155/2023/8567870

**Published:** 2023-02-27

**Authors:** Azar Darvishpour, Shiva Mahdavi Fashtami

**Affiliations:** ^1^Department of Nursing, Zeynab (P.B.U.H) School of Nursing and Midwifery, Guilan University of Medical Sciences, Rasht, Iran; ^2^Social Determinants of Health Research Center, Guilan University of Medical Sciences, Rasht, Iran; ^3^Pirouz Hospital, Guilan University of Medical Sciences, Rasht, Iran

## Abstract

**Background:**

Nurses experience caring burdens, which can affect their caring behaviors. Caring for highly infectious patients, in particular COVID-19, is a new phenomenon and little is known about it. Considering that caring behaviors can be influenced by various factors and cultural differences of the society, it is necessary to conduct studies about caring behaviors and caring burdens. Thus, this study aimed to determine caring behavior and caring burden and their relationship with some associated factors among nurses who cared for patients with COVID-19.

**Materials and Methods:**

This cross-sectional, descriptive design study was conducted by census sampling on 134 nurses working in public health centers in East Guilan, the north of Iran, in 2021. The research instruments included the Caring Behavior Inventory (CBI-24) and the Caregiver Burden Inventory (CBI). Descriptive and inferential statistics were used to analyze the data using SPSS software version 20 with a significant level of 0.05.

**Results:**

The mean score of caring behavior and caring burden in nurses was 126.50 (SD = 13.63) and 43.65 (SD = 25.16), respectively. There was a significant relationship between caring behavior and some demographic characteristics (education, place of living, and history of COVID-19) and between caring burden and some demographic characteristics (housing status, job satisfaction, intention to change job, and history of COVID-19) (*p* < 0.05).

**Conclusions:**

Findings showed that despite the new emergence of COVID-19, the caring burden on nurses was moderate and they had good caring behavior. Despite these results, it is necessary for the relevant managers to pay special attention to protecting health workers during a national crisis such as COVID-19 so that they experience less caring burden and improve caring behavior.

## 1. Background

On 31 December, 2020, an outbreak of pneumonia due to COVID-19 was reported in Wuhan, China [1]. The prevalence of new infectious diseases and emerging dangerous viruses is a global health issue and a threat to nurses and other health care workers [2]. Compared to other health care professions, nurses spend more time with patients and are responsible for direct patient care [3]. Care is one of the basic concepts of nursing [4] and the most important part of nursing practice [5] that provides a framework and guidance for nursing practice [6]. Care can be seen in the form of behaviors [7]. The main goal of caring behavior is to reduce patients' suffering [4]. Caring behaviors are actions concerned with the well-being of a patient, such as sensitivity, comfort, attentive listening, honesty, and nonjudgmental acceptance [8]. Lininger stated caring behaviors include concepts such as comfort, compassion, interest, coping, empathy, facilitation, helpful behaviors, love, nutrition, reinforcement, protective and inspiring behaviors, sharing, helping, support, sensitivity, touch, and trust [9]. Understanding nurses' caring behaviors is very important [6]. Studies on the importance of caring behaviors suggest that caring is not uniformly understood in different communities. Caring behaviors can be influenced by cultural differences. Organizational factors also have the potential to change caring behavior [10]. Differences in nursing care behaviors from one institution to another or from one country to another have caused nursing researchers to study these behaviors [8]. Care-related research and the application of its related results play a vital role in improving and maintaining the quality of nursing care [10].

On the other hand, some studies have shown that nurses experience different care pressures when caring for patients. The caring burden is a type of distress that nurses feel only because they provide patient care and is different from anxiety and depression caused by other emotional issues. The caring burden has physical, psychological, social, and economic dimensions [11] and can cause many problems such as burnout, anxiety, and depression. For caregivers [12], Harding et al. (2015) in a study showed that the caring burden varies for caregivers of patients with cancer, dementia, and brain trauma [13]. This suggests that nurses experience varying degrees of care burden depending on the type of care they provide [11].

Caring behavior might be influenced by different factors including workload, lack of time, staffing issues, shift work, and lack of self-care [8, 14]. A qualitative study conducted by Oskouie et al. in a burn center in Tehran, Iran, reported that personal characteristics of nurses such as conscience, religious beliefs, personal philosophy, sense of responsibility, and altruism may affect nurses' caring behaviors. They also stated that staff shortage, lack of organizational support, heavy workload, low payment, feeling of pressure, lack of motivation, patients' characteristics, and patients' age are the factors that may affect nurses' caring behaviors [15].

Although nursing scholars are unanimous about the fact that caring behaviors might be affected by various factors, not many studies have been carried on to address the determinants of caring behaviors [8]. On the other hand, caring for highly infectious patients is a new phenomenon and little is known about it [16]. In particular, our information and knowledge about COVID-19 and its effects in various fields are low and there is still much to discover. The necessary preparations and measures to deal with it will be possible with the rapid integration of scientific knowledge and public health [17]. In order to deal with the COVID-19 pandemic, it is necessary to draw a clear picture of it [18]. All countries should increase their level of preparedness and response to identify, manage, and monitor new COVID-19 cases [17]. Caring behaviors can be directly influenced by the health care provider, organizational factors, nursing care delivery model, and cultural differences based on common values in society [3]. Therefore, it is necessary that studies on identifying the caring behaviors and caring burden should be conducted in each country, taking into account the existing conditions.

The review of the literature indicates that no study has been conducted on caring behaviors and caring burdens in nurses caring for patients with COVID-19. Considering that recognizing caring behaviors is an essential step in improving caring behaviors and improving the quality of care (especially in patients with COVID-19), ultimately helps to facilitate care planning; this study was conducted to determine caring behavior and caring burden and their relationship with some associated factors among nurses who cared for patients with COVID-19.

## 2. Materials and Methods

### 2.1. Study Design, Setting, and Participants

A cross-sectional, descriptive design study was conducted in 2021. The study setting was the East Guilan public hospitals that were affiliated to Guilan University of Medical Sciences, the north of Iran. In this study, three hospitals out of six public hospitals in East Guilan were selected randomly. A simple random method was used for randomization. In this way, the names of six hospitals were written on small pieces of paper and put inside in an envelope. Then, three small pieces of paper with the name of a hospital written on them were randomly taken out of the envelope. In the three selected hospitals, by using a census sampling, all the nurses working in the COVID and ICU wards who cared for patients with COVID-19 were considered for sampling. The samples included 134 nurses who were recruited based on inclusion criteria. Inclusion criteria included having a bachelor's degree in nursing and higher, working in public hospitals in East Guilan, having experience of caring for a patient with COVID-19, and willingness to participate in the study. Exclusion criteria included the unwillingness to continue cooperation in the study.

### 2.2. Research Instruments

The research instruments included demographic characteristics (e.g., age, sex, level of education, marital status, etc.), the Caring Behavior Inventory (CBI-24), and the Caregiver Burden Inventory (CBI), which are explained below.

#### 2.2.1. Caring Behavior Inventory (CBI-24)

This is an empirical instrument for measuring caring with a clear conceptual-theoretical basis, developed to determine perceptions of caring among patients and nurses in diverse settings [19]. This tool was first designed by Wolf et al. (1981) with 75 items to study caring behaviors in nurses [7]. After revision by Wu et al. (2006), it was reduced to 24 items [20]. This 24-item instrument includes four subscales, namely, (1) the assurance subscale, being readily available to a patient's needs and security (8 items); (2) the knowledge and skill subscale, demonstrating conscience and competence (5 items); (3) the respectful subscale, attending to the dignity of the person (6 items); and (4) the connectedness subscale, providing constant assistance to patients with readiness (5 items) [19]. To measure the average of each subscale, the scores of the items related to each are added and the sum of the scores is divided by the number of items. Each item is based on a six -point Likert scale and is graded from 1 (never) to 6 (always). The minimum score is 24 and the maximum is 144. In this tool, a higher score indicates more appropriate caring behaviors [20]. The caring behavior for each subscale as well as for the overall scale is calculated as the mean value within each separate scale [19]. The internal reliability of the questionnaire in the study by Çelik et al. (2019) and Asadi et al. (2014) was calculated using Cronbach's alpha coefficient of 0.93 and 0.71, respectively [21, 22]. The reliability of the instrument in the present study was calculated using Cronbach's alpha coefficient of 0.79.

#### 2.2.2. Caregiver Burden Inventory (CBI)

This Questionnaire has 24 items, which was developed in 1989 by Novak and Guest. This questionnaire includes five subscales, namely, (1) time dependence (this indicates the amount of time a caregiver spends on taking care of her/his patient) including 5 items (questions 1 to 5); (2) developmental (the pressure/burden that occurs during different periods of the caregiver's life development (such as puberty), caused by taking care of the patient) including 5 items (questions 6 to 10); (3) physical burden including 4 items (questions 11 to 14); (4) social burden including 5 items (questions 15 to 19); and (5) emotional burden including 5 items (questions 20 to 24) [23]. Scoring each dimension includes a five-point Likert scale (0 = not at all; 1 = a little; 2 = medium; 3 = a lot; 4 = a lot). The total score for each dimension is calculated from zero to 20 and the total CBI score from a minimum of zero to a maximum of 100 [24].

The classification of scores is considered as zero to 19 (low caring burden), 20 to 50 (medium caring burden), and 51 to 100 (high caring burden) [11]. Shafizadeh et al. (2017) validated this questionnaire in 150 Alzheimer's patients. The total internal correlation (Cronbach's alpha) for the subscales was 0.93 [25]. The reliability of the instrument in the present study was calculated using Cronbach's alpha coefficient of 0.82.

### 2.3. Data Collection

In order to collect data, the researcher, after obtaining permission from the research ethics committee of Guilan University of Medical Sciences and relevant officials, referred to the medical center. After selecting the samples, providing sufficient explanations about the purpose of the research and obtaining their written consent, the questionnaires were given to the samples to be completed. The samples who were willing to participate in the study signed consent forms. Then, they were given questionnaires and asked to complete them. The completed questionnaires were collected later in the same shift. The data collection period lasted 3 months.

#### 2.3.1. Ethical Consideration

For ethical considerations, permission was obtained from the Research Ethics Committee of Guilan University of Medical Sciences (ethics ID IR.GUMS.REC.1399.648). According to the principles of research ethics, participants were reminded that at each stage of the study, they could refuse to continue their cooperation if they did not want to. They were also reminded that, if they wished, the results of the research would be made available to them and that their information would be kept confidential.

### 2.4. Data Analysis

Data were analyzed using the Statistical Package for the Social Sciences (SPSS), version 20 (IBM Corp., Armonk, NY, USA). The Kolmogorov−Smirnov test was used to investigate the hypothesis of normal data distribution. Data were analyzed using descriptive statistics (frequency distribution tables, mean, and standard deviation) and inferential statistics (*t*-test, ANOVA, and Linear Regression). All calculations were performed considering the significance level (*p* < 0.05).

## 3. Results

Findings related to demographic characteristics showed that half of the participants were in the age group of 21–30 years. The majority were female (93.3%) and married female (83.5%). In terms of education, the majority were bachelors (88.1%) and in terms of work experience, most of them were in the range of 1–5 years of work experience (42.5%). Findings indicated that there is no significant relationship between the caring behavior among nurses who cared for patients with COVID-19 and some demographic variables (age, sex, marital status, income, housing status, number of family members, number of children, responsibility for another person caring, work experience, employment status, shift work, job satisfaction, and intention to change job) (*p* > 0.05). However, there is a significant relationship between the caring behavior and education status, location, and history of COVID-19 (*p* < 0.05). (Table 1).

Findings showed that the mean score of nurses' caring behavior in nurses who cared for patients with COVID-19 was 126.50 (SD = 13.63), which indicated good caring behavior. In addition, the mean score of caring burden in nurses who cared for patients with COVID-19 was 43.65 (SD = 25.16), which indicated the moderate caring burden in nurses (Table 2).

On the other hand, in the relationship between caring behavior and demographic variables due to the presence of five variables (sex, education, place of living, history of COVID-19, and intention to change job) with *p* ≤ 0.2, these variables were allowed to participate in the model regression. In the final stage, history of COVID-19 has the greatest impact on the caring behavior (*p* < 0.05) (Table 3).

The results of the study based on the linear regression model showed that nurses who did not have a history of COVID-19 had a higher mean score of caring behavior (128.40 ± 14.029) than nurses who had a history of COVID-19 (122.73 ± 12.093), and this difference was statistically significant (*t* = 2.311, *p* = 0.02).

Findings indicated that there is no significant relationship between caring burden on nurses who cared for patients with COVID-19 and some demographic variables (age, sex, education, marital status, place of living, income status, number of family members, number of children, responsibility for caring for another person, work experience, employment, and shift work with caring burden) (*p* > 0.05). However, there is a significant relationship between caring burden on nurses who cared for patients with COVID-19 and housing status, job satisfaction, intention to change job, and history of COVID-19 (*p* < 0.05). (Table 4).

In the relationship between caring burden and demographic variables due to the presence of seven variables (education, history of COVID-19, intention to change job, job satisfaction level, income status, housing status, and responsibility for another person caring) with *p* ≤ 0.2, these variables were allowed to participate in the regression model. In the final stage, the factors of the history of COVID-19 and the intention to change job have the greatest impact on caring burden (*p* < 0.05). (Table 5).

The results based on the linear regression model showed that the history of COVID-19 and intention to change job had the greatest impact on the caring burden, so that nurses who did not have a history of COVID-19 had a higher mean score of caring burden (47.04 ± 26.679) compared to nurses with a history of COVID-19 (36.93 ± 20.511). This difference was statistically significant (*t* = 2.230, *p* = 0.027). In addition, nurses who intended to change their jobs had a higher mean score of caring burden (55.06 ± 20.069) than nurses who did not (39.62 ± 25.624), and this difference was statistically significant (*t* = -3.229, *p* = 0.002).

## 4. Discussion

In this study, caring behavior and caring burden and their relationship with some associated factors among nurses who cared for patients with COVID-19 in East Guilan public hospitals were investigated. The results showed that the mean scores of nurses' caring behavior were high and they had good caring behavior. There was not found any similar study in the literature review to discuss the results of this study, and it seems that this study is the first study that deals with this issue. However, to discuss the findings, we tried to use the most relevant evidence for the findings of the present study. Results of a study by Asadi et al. (2014) showed nurses' caring behaviors at the desired level [22]. In addition, results of another study conducted by Çelik et al. (2020), to determine the effect of teamwork attitudes on caring behaviors of nurses working in surgical clinics in a public hospital in Anatolia, Turkey, showed that nurses' perception of caring behaviors was at a good level [21]. Also, Soriano et al. (2019) [6] and Calong and Soriano (2018) [26] reported the nurses' caring behavior was excellent. Optimal status of caring behavior among nurses who cared for patients with COVID-19 in the present study, despite the emergence of this disease and conditions of the country's hospitals with a high workload and nonstandard number of nurses relative to the number of patients [3, 27], indicated high knowledge and professional skills among nurses.

Findings showed that caring burden among nurses who cared for patients with COVID-19 was moderate. The researchers did not find a similar study to discuss our finding. However, the caring burden on caregivers and family members for some patients has been investigated in several previous studies. Results of a study conducted by Ebrahimian et al. (2017) indicated a moderate caring burden [11], which is in line with our findings. Results in another study conducted by Rezaei et al. (2020) showed that the overall percentage of caring burden in patients with chronic disorders was 53.28%. The highest percentages of caring burden were related to dialysis, mental disorders, and Alzheimer's disease, respectively; the lowest percentage of caring burden was related to diabetes [28]. In general, the results of different studies show that caring burden varies in different diseases, and despite the emergence of COVID-19, it was expected that the caring burden on nurses in the present study would be very high. However, the findings indicated that nurses did not experience more caring burden than other disease caregivers, which indicated the good knowledge and readiness of nurses to provide care for all patients, in particular, COVID-19. This finding emphasizes that having knowledge and awareness makes it easier to deal with any type of crisis, even when it is emerging. Therefore, it is necessary and recommended to improve nursing knowledge and practice through continuous education to empower nurses.

Findings of the study indicated that there is no significant relationship between caring behavior among nurses caring for patients with COVID-19 and some demographic variables (age, sex, marital status, income, housing status, number of family members, number of children, responsibility for caring of another person, work experience, employment status, shift work, job satisfaction, and intention to change job). However, there is a significant relationship between caring behavior and education status, the location, and history of COVID-19. In a study conducted by Kotronoulas et al. (2009), there was no significant relationship between nurses' demographic characteristics (age, sex, marital status, clinical work experience, and position) and understanding of the importance of nurses caring behaviors for patients with cancer [29]. In the study conducted by Asadi et al. (2014), the caring behaviors of nurses working in intensive care units were not significantly associated with demographic characteristics (age, sex, marriage, education, and work experience) [22]. Findings in the present study are consistent with the mentioned studies in terms of some demographic characteristics (age, sex, marriage, and work experience). However, our findings are not consistent with them in terms of education. Differences in wards and patients under care can be a justification for this discrepancy. However, higher education leads to a wider range of vision and thinking [30] and can lead to more favorable caring behavior.

In addition, findings in the present study indicated that the history of COVID-19 has the greatest impact on caring behavior. This means that nurses who did not have a history of COVID-19 had a higher mean score for caring behavior than nurses who had a history of COVID-19. This can be due to the effect of COVID-19 on the physical and mental ability of nurses, so that a nurse who is infected with COVID-19, due to the physical and mental problems related to this disease, her/his ability to care is reduced, because herself/himself needs care. Nevertheless, she/he has to go to work and try to meet the needs of her/his patient, and this causes her/his caring behavior to be affected and she/he may not be able to take care of her/his patient, and it disrupts the delivery of her/his caring roles.

Also, findings in the present study indicated that there is no significant relationship between caring burden and some demographic characteristics (age, sex, education, marital status, place of living, income status, number of family members, number of children, responsibility for caring for another person, work experience, employment, and shift work). However, there is a significant relationship between caring burden and housing status, job satisfaction, intention to change job, and history of COVID-19. The caring burden experienced by caregivers can be influenced by many factors [31]. The results of a study showed that age and gender are factors that can greatly affect the caring burden of a patient with dementia [32]. In a study conducted by Talebi et al. (2017), there was a significant relationship between caring burden and age of the caregiver, so that age of the caregiver increases and the caring burden increases [30]. In other studies conducted by Salmani et al. (2014) [33] and Bayoumi (2014) [34], there was a significant relationship between caring burden and age of the caregiver, which is not consistent with the results of the present study. The reason for this discrepancy can be due to the average age of the samples in the present study with the mentioned studies. As the majority of samples (85.1%) in the present study were young and in the range of 21 to 40 years, but the average age of caregivers in the studies was conducted by Talebi et al. (2017), Salmani et al. (2014), and Bayoumi (2014) was 43.42 ± 7.00, 38.41 ± 9.04 and 40 ± 11.00 years, respectively [30, 33, 34]. However, with increasing age, person's physical ability to care for others decreases and the caring burden increases [35], and this can be a justification for the inconsistency of the findings of present study with those studies.

Findings in the present study also showed that the history of COVID-19 and the intention to change job have the greatest impact on caring burden. In this way, nurses who did not have a history of COVID-19 felt a higher caring burden than nurses who had this. In the study by Talebi et al. (2017) [30], there was a statistically significant relationship between the caring burden and the caregiver's illness, so that the caregiver who had the disease herself/himself experienced more caring burden. This finding is not consistent with the results of the present study. In justifying this discrepancy, it can be explained that nurses in the present study who did not have a history of COVID-19 took more protective measures to avoid getting sick and followed health protocols more, and they were under more burden. They were also under pressure to take care of the patients. They may also feel more pressured because of fear of future COVID-19. Therefore, this has led to additional pressure and more caring burden. Regarding the intention of changing job, the results showed that nurses who intended to change their jobs endured a higher caring burden than nurses who did not. It seems they were reluctant to work in this profession due to job dissatisfaction and were forced to attend a work setting and do their job. This forced presence brought them a lot of pressure and stress, it doubled with caring for people with COVID-19, and they felt more caring burden.

The present study, like other studies, has some limitations. The use of questionnaires, which are a self-reporting method for data collection, is not far from being biased in response. Although sampling was conducted with the consent of the staff, the work environment conditions such as workload, stress, and fatigue of the participants may affect the quality of their response. Another limitation was conducting research in public hospitals in East Guilan, which was limited to generalization to other medical centers. It is suggested that future studies should be conducted to clarify the relationship between research variables in the wider statistical population.

## 5. Conclusion

Findings showed that despite the new emergence of COVID-19, caring behavior among nurses who cared for patients with COVID-19 was good, and the caring burden on nurses was moderate. Despite these results, it is necessary for the relevant managers to pay special attention to protecting health workers during a national crisis such as COVID-19, so that they experience a less caring burden and improve caring behavior.

## Figures and Tables

**Table 1:** Demographic characteristics and caring behavior among nurses who cared for patients with COVID-19 in East Guilan public hospitals (*n* = 134).

Demographic variables *n* (%)			Caring behaviors				
			Mean ± SD	Minimum	Maximum	Tests	*p* value
Age (year)	21–30	(50.0) 67	13.73 ± 126.27	92	144	*F* = 1.013	0.468
	31–40	(35.1) 47	13.34 ± 128.32	95	144		
	41–50	(14.9) 20	12.91 ± 124.32	101	144		

Sex	Female	(93.3) 125	13.41 ± 127.06	92	144	*t* = 1.800	0.074
	Male	(6.7) 9	14.99 ± 118.67	100	143		

Marital status	Single/Divorced/widowed	(16.5) 22	13.84 ± 127.80	92	144	*t* = 1.472	0.233
	Married	(83.5) 112	13.46 ± 126.15	95	144		

Number of children	No children	(10.5) 14	11.35 ± 131.00	118	139	*F* = 0.832	0.507
	One	(45.5) 61	14.02 ± 126.03	92	144		
	Two	(35.1) 47	13.69 ± 126.77	97	144		
	Three and above	(8.9) 12	11.21 ± 129.18	113	144		

Place of living	City	(94.8) 127	13.39 ± 126.48	93	144	*t* = 3.988	0.021^*∗*^
	Village/Suburbs	(5.2) 7	11.81 ± 132.67	111	143		

Education^a^	BS	(88.1) 118	13.08 ± 127.42	92	144	*t* = 2.139	0.034^*∗*^
	M.Sc	(11.9) 16	16.019 ± 119.75	95	144		

Employment status^b^	Permanent	(33.6) 45	14.41 ± 123.38	95	144	*F* = 1.316	0.272
	Contracted to permanent	(26.1) 35	11.36 ± 129.14	104	144		
	Contracted	(9.7) 13	14.54 ± 127.77	101	144		
	Graduates' temporary employment contract	(30.6) 41	14.06 ± 127.27	92	144		

Work experience (year)	1–5	57 (42.5)	127.47 ± 13.61	92	144	*F* = 0.556	0.734
	6–10	31 (23.1)	127.65 ± 13.02	103	144		
	11–15	25 (18.7)	123.80 ± 15.39	95	144		
	16–20	13 (9.7)	127.92 ± 12.36	101	143		
	21–25	6 (4)	121.50 ± 15.24	98	144		
	26 and above	2 (1.5)	120.50 ± 6.36	116	125		

Working shifts	Morning	23 (17.2)	122.83 ± 13.442	95	144	*F* = 1.365	0.250
	Night	8 (6.0)	134.00 ± 15.784	101	144		
	Morning and evening	6 (4.5)	124.17 ± 9.948	112	137		
	Rotational	97 (72.4)	126.90 ± 13.576	92	144		

Housing status	Personal	115 (85.8)	125.93 ± 13.63	92	144	*t* = −1.192	0.235
	Rental	19 (14.2)	129.95 ± 13.46	103	144		

Responsibility for another person caring	Yes	7 (5.2)	126.69 ± 13.60	104	144	*t* = 0.696	0.487
	No	127 (94.8)	123.00 ± 14.75	92	144		

History of COVID-19	Yes	45 (33.6)	122.73 ± 12.09	95	144	*t* = 2.311	0.022^*∗*^
	No	89 (66.4)	128.40 ± 14.02	92	144		

Job satisfaction level	Low	68 (51)	126.25 ± 13.20	97	144	*F* = 0.171	0.843
	Medium	57 (42.5)	127.05 ± 13.85	93	144		
	High	9 (6.5)	123.25 ± 21.31	92	140		

Intention to change job	Yes	35 (26.1)	123.37 ± 14.56	95	144	*t* = 1.589	0.115
	No	99 (73.9)	127.61 ± 13.18	92	144		
The number of family members	1–2	35 (26.1)	126.31 ± 12.47	103	144	*F* = 0.004	0.996
	3–4	88 (65.7)	126.57 ± 13.86	92	144		
	≥5	11 (8.2)	126.55 ± 16.41	93	144		

Income status	Low	36 (26.9)	127.14 ± 14.14	100	144	*F* = 1.564	0.213
	Medium	84 (62.7)	127.24 ± 13.12	93	144		
	Sufficient	14 (10.4)	120.43 ± 14.76	92	138		

Note: ^*∗*^significant level is indicated by *p* < .05. ^a^ BS = Bachelor of Science, M.Sc = Master of Science; ^b^ the employment status of Iranian nurses includes Rasmi (Permanent), Paimani (Contracted to Permanent), Gharardadi (Contracted), and Tarhi (Graduates' temporary employment contract), respectively.

**Table 2:** Mean of caring behavior and caring burden among nurses who cared for patients with COVID-19 in East Guilan public hospitals (*n* = 134).

Variable	Mean ± SD	Minimum	Maximum
Caring behaviors	126.50 ± 13.63	92	144
Caring burden	43.65 ± 25.16	0	120

Note: SD = Standard deviation.

**Table 3:** Relationship between variables with a significance level of less than 0.2 (sex, education, place of living, history of COVID-19, and intention to change job) with caring behavior.

Variable	*B*	Std. Error	Beta	*t*	*p* value	95.0% confidence interval for *B*	
						Lower bound	Upper bound
Constant	157.75	8.764	—	18.000	0.000	140.410	175.091
Sex	−7.150	4.562	−0.132	−1.567	0.120	−16.177	1.877
Education	−6.696	3.537	−0.160	−1.893	0.061	−13.694	0.303
Place of living	−4.466	4.290	−0.088	−1.041	0.300	−12.955	4.023
History of COVID-19	−5.145	2.419	−0.179	−2.127	0.035	−9.931	−0.360
Intention to change job	−3.584	2.601	−0.116	−1.378	0.171	−8.730	1.563

**Table 4:** Demographic characteristics and caring burden among nurses who cared for patients with COVID-19 in East Guilan public hospitals (*n* = 134).

Demographic variables			Caring behaviors				
*n* (%)			Mean ± SD	Minimum	Maximum	Tests	*p* value
Age (year)	21–30	(50.0) 67	42.70 ± 22.87	1	96	*F* = 0.337	0.799
	31–40	(35.1) 47	42.94 ± 27.09	0	120		
	41–50	(14.9) 20	49.00 ± 29.08	0	115		

Sex	Female	(93.3) 125	43.66 ± 24.61	0	120	*t* = 0.025	0.980
	Male	(6.7) 9	43.44 ± 33.66	0	94		

Marital status	Single/Divorced/widowed	(16.5) 22	43.54 ± 22.74	0	88	*t* = 1.175	0.312
	Married	(83.5) 112	43.28 ± 26.10	0	120		

Number of children	No children	(10.5) 14	37.00 ± 36.51	0	73	*F* = 1.319	0.266
	One	(45.5) 61	41.43 ± 21.63	0	88		
	Two	(35.1) 47	44.19 ± 27.25	0	120		
	Three and above	(8.9) 12	54.00 ± 32.93	0	115		

Place of living	City	(94.8) 127	44.42 ± 25.20	0	120	*t* = 1.287	0.280
	Village/suburbs	(5.2) 7	31.83 ± 22.46	5	60		

Education^a^	BS	(88.1) 118	42.52 ± 25.92	0	120	*t* = −1.420	0.158
	M.Sc	(11.9) 16	52.00 ± 16.97	19	77		

Employment status^b^	Permanent	(33.6) 45	48.98 ± 26.06	0	120	*F* = 1.526	0.211
	Contracted to permanent	(26.1) 35	44.97 ± 27.95	0	96		
	Contracted	(9.7) 13	37.62 ± 18.82	4	69		
	Graduates' temporary employment contract	(30.6) 41	38.59 ± 22.70	0	88		

Work experience (year)	1–5	57 (42.5)	39.93 ± 22.66	0	88	*F* = 1.289	0.273
	6–10	31 (23.1)	40.68 ± 26.84	0	96		
	11–15	25 (18.7)	47.92 ± 21.63	19	96		
	16–20	13 (9.7)	49.92 ± 27.81	15	120		
	21–25	6 (4)	61.83 ± 31.10	28	115		
	26 and above	2 (1.5)	47.00 ± 66.46	0	94		

Working shifts	Morning	23 (17.2)	45.26 ± 23.76	17	120	*F* = 0.772	0.512
	Night	8 (6.0)	54.25 ± 21.87	29	96		
	Morning and evening	6 (4.5)	34.83 ± 23.88	4	74		
	Rotational	97 (72.4)	42.94 ± 25.84	0	115		

Housing status	Personal	115 (85.8)	41.80 ± 25.29	0	120	*t* = −2.120	0.036^*∗*^
	Rental	19 (14.2)	54.84 ± 21.73	16	96		

Responsibility for another person caring	Yes	7 (5.2)	57.00 ± 22.264	19	85	*t* = −1.448	0.150
	No	127 (94.8)	42.91 ± 25.184	0	120		

History of COVID-19	Yes	45 (33.6)	36.93 ± 20.51	0	73	*t* = 2.230	0.027^*∗*^
	No	89 (66.4)	47.04 ± 26.67	0	120		

Job satisfaction level	Low	68 (51)	49.04 ± 26.13	0	115	*F* = 3.839	0.024^*∗*^
	Medium	57 (42.5)	37.19 ± 22.81	0	120		
	High	9 (6.5)	37.25 ± 20.27	17	65		

Intention to change job	Yes	35 (26.1)	55.06 ± 20.06	1	94	*t* = −3.229	0.002^*∗*^
	No	99 (73.9)	39.62 ± 25.62	0	120		
The number of family members	1–2	35 (26.1)	41.40 ± 27.38	0	115	*F* = 0.705	0.496
	3–4	88 (65.7)	43.53 ± 25.03	0	120		
	≥5	11 (8.2)	51.73 ± 18.09	26	84		

Income status	Low	36 (26.9)	50.94 ± 30.06	0	115	*F* = 2.287	0.106
	Medium	84 (62.7)	41.58 ± 23.32	0	120		
	Sufficient	14 (10.4)	37.29 ± 18.71	1	70		

Note: ^*∗*^significant level is indicated by *p* < .05. ^a^ BS = Bachelor of Science, M.Sc = Master of Sciethe ^b^ the employment status of Iranian nurses includes Rasmi (Permanent), Paimani (Contracted to Permanent), Gharardadi (Contracted), and Tarhi (Graduates' temporary employment contract), respectively.

**Table 5:** Relationship between variables with a significance level of less than 0.2 (education history of COVID-19, intention to change job, job satisfaction level, income status, housing status, responsibility for caring for another person), and caring burden.

Variable	*B*	Std. Error	Beta	*t*	*p* value	95.0% confidence interval for *B*	
						Lower bound	Upper bound
Constant	25.001	17.799	—	1.405	0.163	−10.222	60.224
Education	6.225	6.399	0.081	0.973	0.332	−6.437	18.888
History of COVID-19	−11.731	4.271	−0.221	−2.746	0.007	−20.183	−3.278
Intention to change job	14.042	4.936	0.246	2.845	0.005	4.274	23.810
Job satisfaction level	−4.489	4.065	−0.100	−1.104	0.272	−12.534	3.556
Income status	−4.357	3.682	−0.102	−1.183	0.239	−11.644	2.929
Housing status	9.620	5.959	0.134	1.615	0.109	−2.172	21.413
Responsibility for another person caring	12.660	9.236	0.112	1.371	0.173	−5.617	30.937

## Data Availability

The data set generated in this study is available upon reasonable request from the corresponding author.
